# Charge neutralized poly(β-amino ester) polyplex nanoparticles for delivery of self-amplifying RNA†

**DOI:** 10.1039/d3na00794d

**Published:** 2024-01-24

**Authors:** Nazgol Karimi Dastgerdi, Nurcan Gumus, Hulya Bayraktutan, Darryl Jackson, Krunal Polra, Paul F. McKay, Fatemeh Atyabi, Rassoul Dinarvand, Robin J. Shattock, Luisa Martinez-Pomares, Pratik Gurnani, Cameron Alexander

**Affiliations:** a Division of Molecular Therapeutics and Formulation, School of Pharmacy, University of Nottingham NG7 2RD UK cameron.alexander@nottingham.ac.uk; b Department of Pharmaceutical Nanotechnology, School of Pharmacy, Tehran University of Medical Sciences Tehran Iran; c School of Life Sciences, Faculty of Medicine & Health Sciences, University of Nottingham NG7 2RD UK; d Department of Infectious Diseases, Section of Immunology of Infection, Imperial College London Norfolk Place London W21PG UK; e Nanotechnology Research Centre, Faculty of Pharmacy, Tehran University of Medical Sciences Tehran 1417614315 Iran; f UCL School of Pharmacy, University College London 29-39 Brunswick Square London WC1N 1AX UK p.gurnani@ucl.ac.uk

## Abstract

Therapeutic self-amplifying RNA (saRNA) is a promising approach for disease treatment, as it can be administered in lower doses than messenger RNA (mRNA) to achieve comparable protein production levels. However, saRNA requires an appropriate delivery vehicle to protect it during transit and facilitate its transfection. A widely-adopted approach has been to use polycations to condense these large anionic macromolecules into polyplex nanoparticles, however their high charge density often elicits cytotoxic effects. In this study we postulated that we could improve the potency and tolerability of such delivery vehicles by co-formulating poly(β-amino ester)s saRNA polyplexes with a non-toxic anionic polymer, γ-polyglutamic acid (γ-PGA) to neutralize partially this positive charge. Accordingly, we prepared a poly(β-amino ester) from 1,6-hexanedioldiacrylate (HDDA) and 4-aminobutanol (ABOL) and initially evaluated the physicochemical properties of the binary polyplexes (*i.e.* formed from polymer and saRNA only). Optimised binary polyplex formulations were then taken forward for preparation of ternary complexes containing pHDDA–ABOL, saRNA and γ-PGA. Our findings demonstrate that γ-PGA integration into polyplexes significantly enhanced transfection efficacy in HEK293T and A431 cells without affecting polyplex size. Notably, γ-PGA incorporation leads to a pronounced reduction in zeta potential, which reduced the toxicity of the ternary complexes in moDC, NIH3T3, and A431 cells. Furthermore, the presence of γ-PGA contributed to colloidal stability, reducing aggregation of the ternary complexes, as evidenced by insignificant changes in polydispersity index (PDI) after freeze–thaw cycles. Overall, these results suggest that incorporating the appropriate ratio of a polyanion such as γ-PGA with polycations in RNA delivery formulations is a promising way to improve the *in vitro* delivery of saRNA.

## Abstract 2

Therapeutic self-amplifying RNA (saRNA) is a promising approach for disease treatment, as it can be administered in lower doses than messenger RNA (mRNA) to achieve comparable protein production levels.

## Graphical abstract

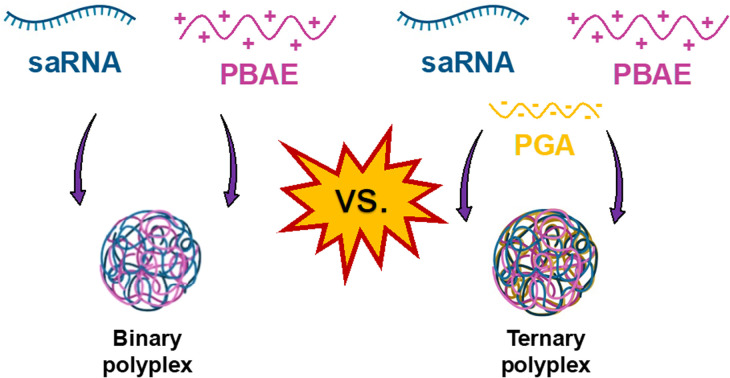

## Introduction

The use of messenger RNA (mRNA) as a therapeutic agent offers many possibilities for treating a wide range of diseases. In principle, mRNA can encode any protein of interest, and because RNA therapies do not require penetration into the nucleus for the transgene to be expressed, transfection is generally more successful for RNA systems compared to DNA-based analogues.^1^ In addition, mRNA can now be prepared quickly and easily by standardised *in vitro* transcription techniques, so its wider-scale manufacture is relatively simple and rapid, in comparison with protein production.^2,3^ Accordingly, in addition to the Pfizer-BioNTech and Moderna COVID-19 vaccines, there are many other RNA vaccines and therapeutics under development.^4^ Despite the successful implementation of mRNA into vaccine formulations, there remain challenges to decrease mRNA content in the formulations to reduce dose-associated side effects and overall cost.^5^

One such strategy is the development of self-amplifying RNA (saRNA) that not only encodes for the protein of interest, but also includes replicase enzymes which enable the administered RNA to be amplified within the cytosol reducing the overall dose requirements and significantly lengthening the expression half-life.^6^ For instance self-amplifying RNA vaccines have displayed similar levels of protective immunity at 100-fold lower doses compared to analogous mRNA constructs to reach the same protein expression.^7,8^ However, the delivery obstacles for saRNA delivery remain essentially the same as those for mRNA, including susceptibility to enzymatic degradation, rapid renal clearance, and poor cell membrane penetration.^7–13^ In addition, the larger size of saRNAs (typically 9500 nt) compared to traditional mRNAs (∼2000 nt), may also alter the mechanisms of condensation with polycations and in addition increase their potential for chain cleavage which deactivates their expression. These obstacles necessitate the use of a delivery vehicle to protect the RNA cargo, aid transport across to the target tissue, penetration of the cell membrane and entry into the cytosol.

Of these delivery systems, many non-viral vectors have been reported for mRNA delivery, with lipid nanoparticles (LNPs) exclusively used for both mRNA and saRNA formulations.^14,15^ Nevertheless, LNPs have some non-optimised properties such as difficult sterilization processes, and degree of susceptibility to oxidation. In addition, the LNPs chemical space is highly protected in terms of intellectual property (IP) which may hinder certain commercial applications.^16^ Polymeric vectors have potential as alternatives to lipids for RNA delivery owing to their wide chemical diversity, formulation flexibility, range of functional groups for targeting, stability in polyplex form, and well-established manufacturing processes.^17^ Indeed, many different cationic polymers have been used as non-viral polymeric vectors for RNA delivery, including poly(ethyleneimine),^18^ poly(lysine),^19^ chitosan,^20^ polyamidoamines^21^ and poly(β-aminoesters).^22^

Although polymer–RNA complexes are widely established in this field, the high positive charge density of these materials means that there are toxicity concerns potentially limiting clinical applications. A high cationic charge may elicit adverse effects due to pain on injection, cell membrane damage, off-target distribution or elimination *via* nonspecific protein absorption in the bloodstream.^16^ One possible strategy is to use negatively charged polymers to reduce charge–charge interactions with lipid membranes and thus reduce toxicity, and to modulate intracellular processing to improve transfection efficacy. It is also postulated that penetration through tissue matrices might be improved *via* reduction in polyplex surface cationic charge.^23,24^ In prior work, Hsu *et al.* showed that a combination of cationic side-chain poly(phosphazene)s with pDNA and siRNA in ternary complexes with anionic side-chain poly(phosphazene)s displayed higher transfection efficacy compared to polycation-only polymer/pDNA and siRNA complexes.^25^ More recently, Hachim *et al.* showed that polysaccharide-based polyanions can enhance transfection efficacy in-polyelectrolyte nanofilms.^26^ However, this approach has, to our knowledge, not been applied to saRNA which, due to its length, has subtly different formulation requirements to pDNA, mRNA and also to siRNA. We were particularly interested to evaluate if simple poly(carboxylic acid)s could be added to polycation/saRNA mixtures to reduce the net surface charges of complexes containing an excess of polycation compared to RNA, yet retain stability sufficient for delivery and transfection.

Here, we demonstrate the enhanced delivery of saRNA *in vitro* through the use of charge-neutralized polyplexes based on a poly(β-amino ester)s (PBAE) platform co-formulated with a range of poly(glutamic acid) (PGA) analogues, a negatively charged biodegradable polypeptide.^27,28^ We describe the synthesis of the poly(β-amino ester) from 1,6-hexanedioldiacrylate (HDDA) and 4-aminobutanol (ABOL) and subsequent optimisation and characterisation of this base pHDDA–ABOL polyplex, which is prepared with saRNA. The effect of PGA structure (α- and γ-PGA), molar mass and incorporation methods were tested to identify the most suitable PGA analogue. The impact of formulation properties on the size, surface charge, entrapment efficacy and stability of the delivery system was evaluated. Finally, the internalisation and saRNA targeting of the nanocomplexes was evaluated in several relevant cell lines *via* flow cytometry and confocal microscopy to determine the potential of these formulations for saRNA delivery.

## Materials and methods

### Materials

All solvents were of analytic or HPLC grade and purchased from Sigma Aldrich or Fisher Scientific unless otherwise stated. All deuterated solvents were purchased from Sigma Aldrich. 1,6-Hexanediol diacrylate, 4-amino-1-butanol and γ-poly(l-glutamic acid) were purchased from Sigma-Aldrich. α-Poly(l-glutamic acid) 3.5 kDa and 18.5 kDa were purchased from Iris BioTech GmbH.

### Instrumentation

#### Size exclusion chromatography (SEC)

The molar mass (*M*_n,SEC_) and dispersity (*Đ*) were characterized using a Polymer Laboratories PL-50 instrument, equipped with a differential refractive index (DRI) detector, with dimethylformamide (DMF) containing 0.1 wt% LiBr as the eluent at a flow rate of 1.0 mL min^−1^ with the column heating to 50 °C. Two columns consisting of 2 × PLgel Mixed D columns (300 × 7.5 mm) and a PLgel 5 μm guard column were used in series. Poly(methyl methacrylate) standards were used for instrument calibration. The polymers were dissolved in DMF, containing 0.1 wt% LiBr, and filtered prior to the injection.

#### ^1^H NMR spectroscopy

^1^H NMR spectra were recorded using a Bruker AV 400 MHz spectrometer at room temperature in deuterated solvents. Measurements were recorded as an average of 16 scans.

#### Particle size and zeta potential

Dynamic light scattering (DLS) was performed to measure the size distribution of the polyplexes and ternary complexes by using Malvern Zetasizer Nano-ZS (Malvern Inst. Ltd., Malvern, UK). The analysis was performed with a detection angle of 90 °C and at the wavelength of 633 nm at 25 °C. Aliquots (500 μL) of the sample were mixed with the same volume of NaCl solution (5 mM) for measuring the zeta potential. The zeta potential values were measured by Laser Doppler Electrophoresis using a Zetasizer (Nano-ZS, Malvern, UK).

#### Transmission electron microscopy (TEM)

A fresh suspension of the polyplexes and ternary complexes was prepared in nuclease free water to be imaged by TEM. A drop of sample was placed onto a carbon film grid with 200 mesh copper (EMResolution, UK). After removing the excess solution, the samples were stained with 2% (w/w) uranyl acetate, washed with DI H_2_O twice and allowed to air-dry. Samples were then imaged on a TEM-2100 Plus electron microscope (JEOL USA, Peabody, MA, USA) using a voltage of 100 kV.

### Methods

#### Synthesis of pHDDA–ABOL polymer

1,6-Hexanediol diacrylate (HDDA, 246816 Sigma Aldrich) and 4-amino-1-butanol (ABOL, 178330 Sigma Aldrich) were dissolved in DMSO to prepare stock solutions at 500 mg mL^−1^ in volumetric flasks. Stock solutions of 1.12 mL containing 0.4 mL of the HDDA (0.56 g, 2.47 mmol) and ABOL (0.2 g, 2.24 mmol) were mixed in a 4 mL borosilicate glass test tube equipped with a 1 cm magnetic stirrer. The tube was sealed with an appropriately sized rubber septum, covered with aluminium foil and immersed in an oil bath preheated to 90 °C. After 48 h the mixture had turned a clear orange-brown colour and was removed from the oil bath then cooled to room temperature. A 100 μL sample of the polymerisation mixture was taken for analysis. For end-capping, the polymerisation mixture was diluted to 150 mg mL^−1^ polymer with DMSO and ABOL was added such that the final concentration was 0.5 M. A further 100 μL sample was taken for post-capping characterisation. The polymer was isolated by three repeated precipitations and centrifugation cycles in ice-cold diethyl ether and dried under reduced pressure to yield pHDDA–ABOL as a viscous orange-brown oil. The final polymer was characterised by ^1^H NMR spectroscopy and size exclusion chromatography.

#### FITC labelling of pHDDA–ABOL

1 mg (1.140 × 10^−3^ mmol) of fluorescein isothiocyanate (FITC) was dissolved in 100 μL of DMF. Then the FITC solution was added to 250 μL of the pHDDA–ABOL stock solution (100 mg mL^−1^) in DMSO. The reaction was conducted in the dark at 25 °C for 24 h. After completion of the reaction, the sample was dialysed against sodium acetate 25 mM (500 mL) with a pH of 5.5 in Float-a-lyzer dialysis tubes (molecular weight cut-off = 1 kDa) for 48 h. The sodium acetate was replaced every 6 h. Finally, the labelled polymer was isolated after freeze-drying.

#### RNA *in vitro* transcription and purification

saRNA was prepared as previously described.^29^ Briefly, self-amplifying RNA encoding the replicase derived from the Venezuelan Equine Encephalitis Virus (VEEV) and either firefly luciferase (fLuc) or enhanced green fluorescent protein (eGFP) was produced using *in vitro* transcription. pDNA was transformed into *E. coli*, cultured in 100 mL of Lysogeny Broth (LB) with 100 μg mL^−1^ carbenicillin (Sigma Aldrich, UK) an isolated using a Plasmid Plus MaxiPrep kit (QIAGEN, UK). The concentration and purity of pDNA was measured on a NanoDrop One (ThermoFisher, UK) and subsequently linearized using MluI for 3 h at 37 °C. For *in vitro* transfections, capped RNA was synthesized using 1 μg of linearized DNA template in a mMessage mMachine™ (Ambion, UK) and purified using a MEGAClear™ column (Ambion, UK) according to the manufacturer's protocol. Uncapped RNA transcripts were synthesized using 1 μg of linearized DNA in a MEGAScript™ reaction (Ambion, UK) according to the manufacturer's protocol. Transcripts were then purified by overnight LiCl precipitation at −20 °C, pelleted by centrifugation at 14 000 rpm on tabletop centrifuge for 20 min at 4 °C, washed 1× with 70% EtOH, centrifuged for 14 000 rpm for 5 min at 4 °C, and then resuspended in UltraPure H_2_O (Ambion, UK). Purified transcripts were then capped using the ScriptCap™ Cap 1 Capping System Kit (CellScript, Madison, WI, USA) according to the manufacturer's protocol. Capped transcripts were then purified by LiCl precipitation as detailed above, resuspended in UltraPure H_2_O and stored at −80 °C until further use.

### Polyplex formulation and characterisation

#### Binary polyplex formulation

Binary polyplexes were prepared using the following general procedure. A stock solution of saRNA (10 μg mL^−1^) was diluted in 10 mM HEPES (pH = 7.4, prepared in nuclease free ultrapure water) corresponding to different desired N : P ratios to obtain the polyplexes. An equal volume of the pHDDA–ABOL polymer solution (1.92 mg mL^−1^) was prepared in 10 mM HEPES (pH = 7.4, prepared in nuclease free ultrapure water) and transferred to an appropriate sized vial. The saRNA solution was loaded in a syringe and infused *via* a syringe pump infusion over 1 min onto the pHDDA–ABOL solution with stirring (1000 rpm). Polyplexes were left to form over 10 min and used directly.

#### Ternary polyplex formulation

The pHDDA–ABOL:PGA:saRNA complexes were prepared with two different methods. In the first method, the mixture of the saRNA solution (500 μL, 15 μg mL^−1^) and different concentrations (7.3, 18.5, 36.9, 73.9, 147.8 μg mL^−1^) of the PGA (500 μL) were added by syringe pump (flow rate 0.5 mL min^−1^) to pHDDA–ABOL (500 μL, 1.92 mg mL^−1^) with the stirring in the receiver flask (1000 rpm) to prepare the ternary complex with desired N : C : P ratio. In the second method, the pre-prepared polyplexes were coated with PGA. The first step in this process was to prepare polyplexes. The saRNA solution (500 μL, 0.3 μg mL^−1^) was loaded into the syringe, and it was added to the pHDDA–ABOL solution (500 μL, 100 mg mL^−1^) with a flow rate of 0.5 mL min^−1^. Then the mixture was mixed at 1000 rpm to prepare pHDDA–ABOL:saRNA polyplexes. After that, the appropriate concentration of PGA (in a volume of 500 μL) was added to the prepared polyplex by syringe pump to form the coated polyplex.

#### RiboGreen encapsulation efficiency

The RiboGreen assay was performed following the procedures. Initially, the Quant-iT Ribogreen reagent was diluted with Tris–EDTA buffer (200 mM Tris–HCl, 20 mM EDTA, pH 7.5 in DEPC-treated water) (1×) at a 1000-fold ratio. Then, equal volumes of the diluted Ribogreen reagent (100 μL) and the samples (100 μL) were combined in a 96 flat black well plate (Nunclon). After a 20 min incubation period, the fluorescence of the RiboGreen-bound samples was measured using a Tecan plate reader, with an excitation wavelength (*λ*_ex_) of 480 nm and an emission wavelength (*λ*_em_) of 520 nm. The saRNA encapsulation efficiency was measured by comparing the fluorescence values of the samples to that of negative control.

#### Freeze–thaw stability assay

Samples were frozen at −20 °C for a duration of 24 h. Following the freezing period, the samples were thawed at room temperature. Once thawed, further characterisation by dynamic light scattering was performed.

### *In vitro* studies

#### General cell culture

HEK293T, A431 and NIH 3T3 cells (ATCC, USA) were cultured in Dulbecco's Modified Eagle Medium (DMEM, Thermo Scientific, 10566016) with high glucose (4.5 g L^−1^ glucose) supplemented containing 10% fetal bovine serum (FBS), and 1 mM l-glutamine (Thermo Scientific, 25030149). Cells were plated to a 96-well clear flat bottom plate at a cell density of 7000 cells per well and allowed to attach for 24 h at 37 °C, 5% CO_2_ in a humidified incubator to reach 90% confluency.

Human monocytes were isolated from peripheral blood mononuclear cells (PBMCs) obtained from buffy coats (Blood Transfusion Service, Barnsley) using Histopaque 1077 (Sigma, 10771–100 mL) density gradient centrifugation followed by positive selection using CD14+ beads (Miltenyi, 130-050-201). Monocytes were re-suspended in complete RPMI medium (Roswell Park Memorial Institute (RPMI) 1640, Sigma, R0883), 10% (v/v) human AB serum (Sigma, H4522-100ML), 2 mM Glutamax (Sigma, 35050-038), 10 mM HEPES (Gibco, 15630056) with 50 ng mL^−1^ each IL-4 (Miltenyi, 130-093-922) and GM-CSF (Miltenyi, 130-093-866) and incubated in Tissue culture-treated plates (Corning Costar, 3524) for 6–7 days with feeding with complete RPMI medium with cytokines on day 3 at 37 °C, 5% CO_2_. Differentiated cells were harvested by incubating on ice for 30 min and collected by gentle pipetting. Cells were counted and diluted to required cell densities for each experiment and stored on ice until needed. For cell counting, cells were mixed with 0.4% Trypan blue (1 : 1 dilution, Sigma, T8154-100ML) and counted using a TC20™ automated cell counter (Bio-Rad).

#### Transfection with saRNA polyplexes

The cells were plated in a clear 96 well-plate as mentioned above. After that, the medium was aspirated, and replaced with 180 μL of pre-warmed Opti-MEM (Thermo Scientific, 31985070 (https://www.thermofisher.com/order/catalog/product/31985070)). Then cells were incubated for 4 h with 20 μL of the pHDDA–ABOL:PGA:saRNA complexes at 100 ng per well saRNA (1 μg mL^−1^ saRNA) in Opti-MEM for 4 h at 37 °C, 5% CO_2_. At 4 h post-transfection, the transfection media was replaced with fully supplemented medium (cDMEM for HEK293T, A431 and NIH 3T3 cells and RPMI for moDC) and cells were incubated for 24 h at 37 °C, 5% CO_2_ in a humidified incubator. Subsequently, the luciferase activity for each well was determined using Luciferase reagent (ONE-Glo) as follows; 50 μL of the ONE-Glo reagent was mixed with the same amount of the supernatant from the cell. After 10 min incubation at 37 °C, the samples were transferred to a 96 white well-plate and the luciferase activity was determined by measuring the luminescence on a TECAN plate reader. Untreated cells and lipofectamine complex-treated cells were used as a negative control and the benchmark positive control, respectively.

The lipofectamine:saRNA complex were prepared by adding the saRNA solution to the diluted lipofectamine following the manufactures protocol. Initially, a 0.15 μL lipofectamine solution of was diluted with 0.75 μL of Opti-MEM medium. After 3 min of vortexing, the mixture was incubated for 10 min. Next, the saRNA solution was added to the lipofectamine mixture and gently mixed (the final saRNA concentration was 100 ng per well). After a 5 min incubation period, the resulting complex was added to the cells.

#### Cytotoxicity assay

The cytotoxicity analysis was performed as above but 24 h after treating the cells. The medium in each well was replaced with 100 μL of 10% PrestoBlue in Phosphate-Buffered Saline (PBS). After incubation at 37 °C and 5% CO_2_ for 30 min, the fluorescence intensity was measured at 560 nm excitation and 590 nm emission to determine the metabolic activity of the cells. Cells treated with 1% Triton X-100 were used as a positive control group. The metabolic activity (%) measurement was calculated based on the equation below where RFU is relative fluorescence units.
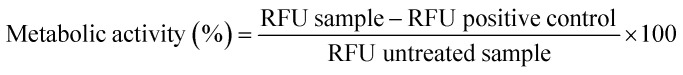


#### Confocal imaging

HEK293T, A431, NIH 3T3 and moDC cells were plated on glass-bottom dishes (35 × 10 mm) at a density of 10^5^ cells/dish 24 h before transfection. The cells were incubated with 100 μL of FITC-labelled pHDDA–ABOL polymer ternary complexes in 900 μL of Opti-MEM. After 4 h post transfection, the lysosomes were stained with 0.4% (v/v) red lysotracker (ThermoFisher, UK) in PBS for 30 min at 37 °C, 5% CO_2_. Then the cells were washed twice with PBS and incubated with Hoechst 33342 (10 μg mL^−1^) for 20 min to stain the nucleus. The cells were washed twice with PBS before imaging on a Zeiss LSM880 confocal microscope (ZEISS, Germany) using ZEN black software (ZEISS, Germany). Images were processed using ImageJ (NIH, USA).

#### Flow cytometry

HEK293T, A431, NIH 3T3 and moDC cells were transfected with FITC-labelled samples as described above. After 4 h incubation, the transfection medium was removed, and the cells were washed twice with PBS. The cells were trypsinised and centrifuged at 1750 rpm for 5 min. The cells were resuspended in 100 μL of PBS containing 5% FBS and analysed with an ImageStream X MKII (Luminex) flow cytometer utilising a 40× objective and 488 nm laser set to 3 mW. Channel 2 (bandpass filter 528/65 nm) was used for FITC fluorescence. Data were analysed using IDEAS Software (V6.2 Luminex). The distribution of labelling was analysed using the Internalisation feature. Briefly, an internal mask was created on the brightfield image by eroding the object mask by 5 pixels and the fluorescence intensity was examined. The internalisation feature reports the ratio of internal fluorescence to total cellular fluorescence mapped to a log scale such that cells with high internal fluorescence have a positive score, those with balanced fluorescence have a score around zero and those with little internal fluorescence have a negative score.

## Results

### Polymer synthesis and characterization

**Fig. 1:**
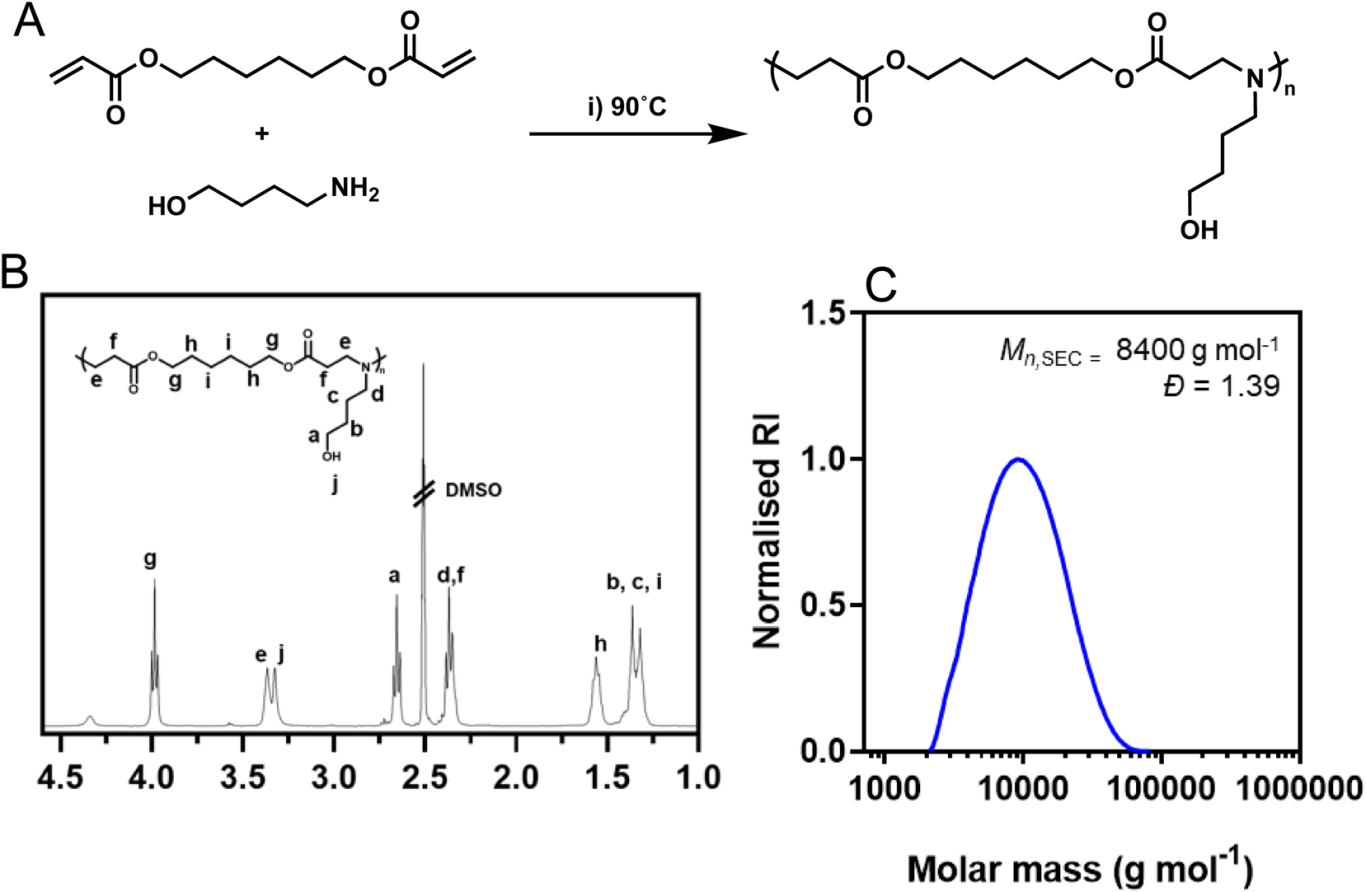
Synthesis of pHDDA–ABOL and physical characterisation. (A) Synthetic scheme to prepare pHDDA–ABOL from HDDA and ABOL monomers. (B) ^1^H NMR spectrum of pHDDA–ABOL recorded in DMSO-d_6_. (C) DMF-SEC chromatogram of pHDDA–ABOL.

Poly(β-amino ester) based formulations have demonstrated significant potential as effective carriers for delivering nucleic acids.^30^ Initially, our polycation of choice, a poly(β-amino ester) (PBAE) based on 1,6-hexanediol diacrylate and 4-amino-1-butanol was synthesized using conventional aza-Michael addition chemistries using a diacrylate : amine ratio of 1.1 : 1 to ensure post-functionalisable acrylate end groups were present (Fig. 1A). The polymerization was performed at high concentration (500 mg mL^−1^) to yield higher molar mass polymers which have previously been shown to improve transfection efficiency of nucleic acids.^29^ Following synthesis, the acrylate end groups were postmodified with 4-amino-1-butanol to match the repeating unit of the polymer using a subsequent aza-Michael addition. The structure of the synthesized polymer was confirmed through ^1^H NMR spectroscopy (Fig. 1B) and SEC analysis pHDDA–ABOL revealed unimodal molar mass distributions (*M*_n,SEC_ = 8400 g mol^−1^, *Đ* = 1.39; Fig. 1C) as expected from such poly(β-amino ester) preparations.

### Optimisation of binary polyplex formulation

**Fig. 2:**
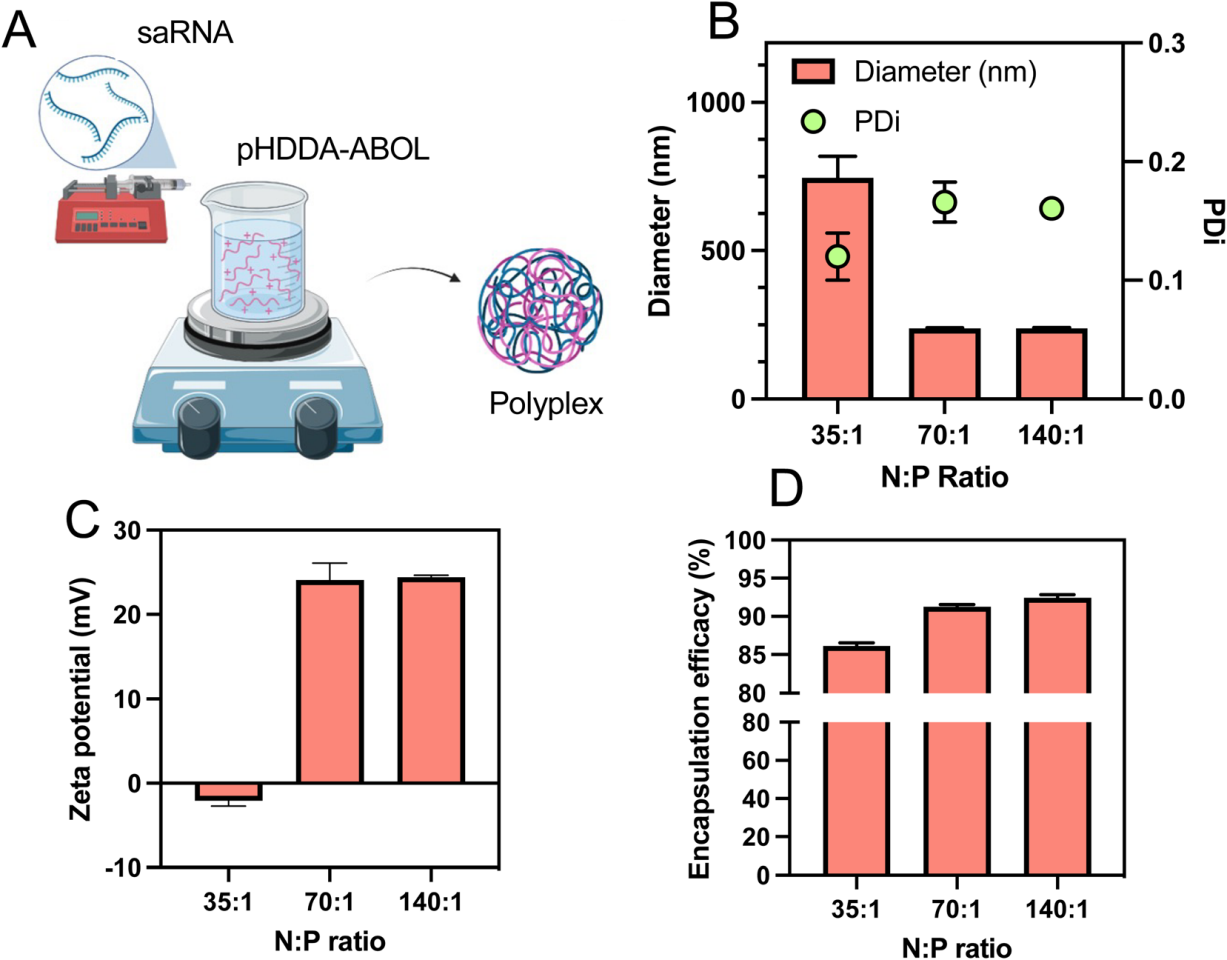
Physicochemical properties of pHDDA–ABOL:saRNA polyplexes. (A) Formulation of binary pHDDA–ABOL and saRNA polyplexes and (B) particle size and PDI, (C) zeta potential, and (D) saRNA encapsulation efficiency of the pHDDA–ABOL:saRNA polyplexes with different N : P ratios (35 : 1, 70 : 1, 140 : 1).

Formulations containing pHDDA–ABOL:RNA polyplexes (binary polyplex) were evaluated initially for key physicochemical characteristics required for saRNA delivery to optimise the polycation : polyanion ratio, usually expressed as nitrogen : phosphate or N/P ratio, which is known to influence the transfection efficacy of nucleic acids.^31^ A previous study used a syringe pump system to mix solutions of polycation and RNA and form the polyplex nanoparticles in a semi-batch process.^32^ We adopted this preparation method due to its ability to generate appropriately-sized polyplexes in a reliable and reproducible manner. Accordingly, to prepare polyplexes the saRNA solution was loaded in the syringe and slowly (0.5 mL min^−1^) injected into the pHDDA–ABOL polymer solution, after which the mixture was stirred at high speed (1000 rpm) (Fig. 2A). Also, various ratios of the polymer to saRNA (N : P ratio where N represents the content of protonatable amines in one polymeric unit of cationic polymer (pHDDA–ABOL) and P represents the content of phosphates within the saRNA backbone) were used to form these polyplexes. After that, the polyplexes were characterised to screen the formulation space and identify potent complexes. Based on previous reports we tested the range of N : P ratios of 35 : 1, 70 : 1, and 140 : 1. We found that increasing the N : P ratio from 35 to 140 reduced the average size of the polyplexes to below the typical threshold value of 200 nm. The observed low polydispersity index (PDI) for all of the polyplexes (≤0.15) illustrated a narrow distribution of polyplex sizes (Fig. 2B). The zeta potentials of the polyplexes increased from −2.1 to 24.1 mV by adding more cationic polymer as expected (Fig. 2C). TEM micrographs (Fig. 4D) demonstrated that the polyplexes were spherical in shape. The association of saRNA with pHDDA–ABOL was measured by the RiboGreen assay, and this revealed that saRNA was almost fully encapsulated in all of the polyplexes (entrapment efficacy > 86%). A rise in the entrapment efficacy was observed when the N : P ratio increased from 35 : 1 to 140 : 1, with N : P ratio of 140 : 1 the saRNA entrapment reached 92% (Fig. 2D). These initial experiments suggested that polyplex preparation for subsequent transgene expression studies should be carried out at an N : P ratio of 140 based on the size, narrow size distribution, positive surface charge, and high incorporation of saRNA at this polymer content.

### Comparison of polyglutamic analogues and mode of incorporation

**Fig. 3:**
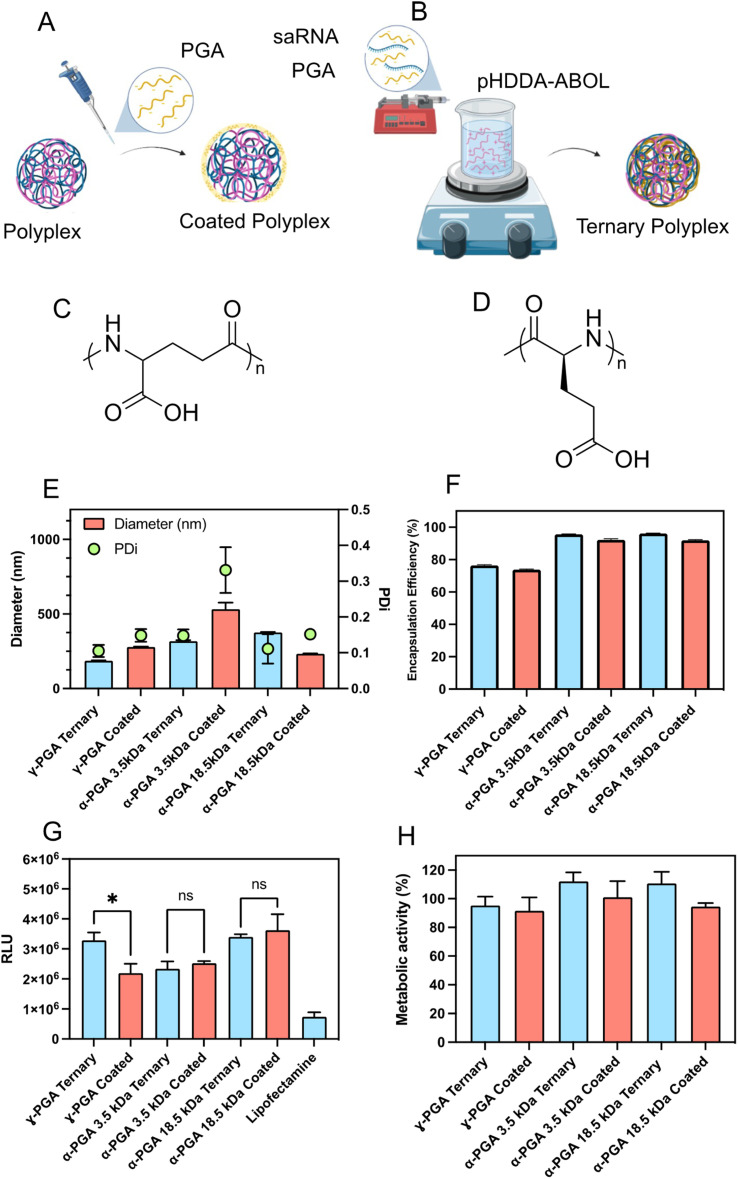
Assessment of different isomers and molar mass poly(glutamic acid) ternary and coated polyplexes. Schematic representation of polyplex preparations for (A) coated polyplexes and (B) ternary polyplexes. Chemical structures of (C) γ-poly(glutamic acid) and (D) α-poly(glutamic acid). Effect of molecular weight and PGA isomer in ternary polyplexes at an N : C : P ratio of 140 : 10 : 1 on (E) particle size and PDI, (F) RNA encapsulation efficiency, (G) transfection efficiency and (H) metabolic activity.

The presentation of high positive charges at the surfaces of injected nanoparticles is a potential problem for delivery due to adsorption of proteins in tissue or plasma and subsequent targeting for elimination of the nanoparticles by the reticulo-endothelial system (RES). One solution to this is to add negatively-charged polymers to the polyplexes, but this requires careful formulation in order not to destabilise the interactions of the cationic polymer with RNA. We thus studied how the properties of the complexes were affected by co-formulating with-poly(glutamic acid) (PGA), a readily-available and non-toxic polyanion. However, due to the presence of two carboxylate moieties, poly(glutamic acid) exists as two possible isomers, α-polyglutamic acid and γ-polyglutamic acid which may yield different activities. Hence we initially screened three different PGA variants, γ-PGA (*M*_w_ = 15–50 kDa), and a high (*M*_w_ = 18.5 kDa) and low (*M*_w_ = 3.5 kDa) molar mass α-PGAs (Fig. 3C and D). The PGAs were incorporated using two methodologies, either PGAs were added to pre-formed pHDDA–ABOL:saRNA binary polyplexes to produce PGA coated polyplexes (Fig. 3A), or a mixture of the negatively charged materials *i.e.* saRNA and PGA were directly co-formulated with pHDDA–ABOL solution to yield ternary complexes (Fig. 3B). Formulations were constructed based on charge ratios 140 : 10 : 1 expressed as an N : C : P ratio (C represents carboxylate) to account for the further negative charge incorporated *via* the PGA.

The experimental data indicates that incorporation of γ-PGA and low MW (molecular weight) α-PGA decreases the size of such ternary complexes when compared to the coated polyplexes. Of particular interest, the ternary complex containing γ-PGA demonstrated the smallest size, below 200 nm in diameter. Additionally, the Ribogreen assay show that all of the formulated complexes demonstrated acceptable entrapment efficacy levels above 70% (Fig. 3F). Remarkably, it was observed that all ternary complexes exhibited a substantial increase in saRNA entrapment compared to their coated polyplexes suggesting that the dynamics of the initial assembly play a role in mediating the overall charge neutralization (Fig. 3E).

The saRNA transfection efficiency and acute toxicity of the coated and ternary complexes were evaluated in HEK293T cells. The γ-PGA ternary complexes significantly enhanced luciferase transgene expression compared to the analogous γ-PGA-coated polyplexes. Additionally, the γ-PGA ternary complex markedly improved transfection efficacy compared with the low molar mass α-PGA ternary complexes and coated polyplexes, meanwhile there was no significant difference in the transfection efficacy of γ-PGA ternary complexes and the formulations containing high molar mass α-PGA. Furthermore, the higher molar mass α-PGA complexes transfected the HEK293T cells more effectively than low molar mass α-PGA. Incorporating PGA in all the formulations enhanced the transfection in comparison with lipofectamine by at least two-fold (Fig. 3G). Additionally, no significant cellular toxicity was shown in any of the formulations after using the negatively charged polymer (Fig. 3H). These results indicate that the α-PGA complexes exhibit no preference for ternary or coated formulation approaches, while γ-PGA formulations exhibited a 50% improvement when co-formulated directly. PGA molar mass was a major driver in transfection efficiency, with both the higher molar mass α-PGA (18.5 kDa) and γ-PGA, (15–50 kDa) exhibiting higher luciferase expression than the 3.5 kDa α-PGA. Both features may be due to the differences in local p*K*_a_ either from chain length or carboxylate position. Following these findings, we therefore opted to continue with γ-PGA to further optimise these formulations.

### Optimisation of polyglutamic acid content

#### Physicochemical properties of polyglutamic ternary complexes

**Fig. 4:**
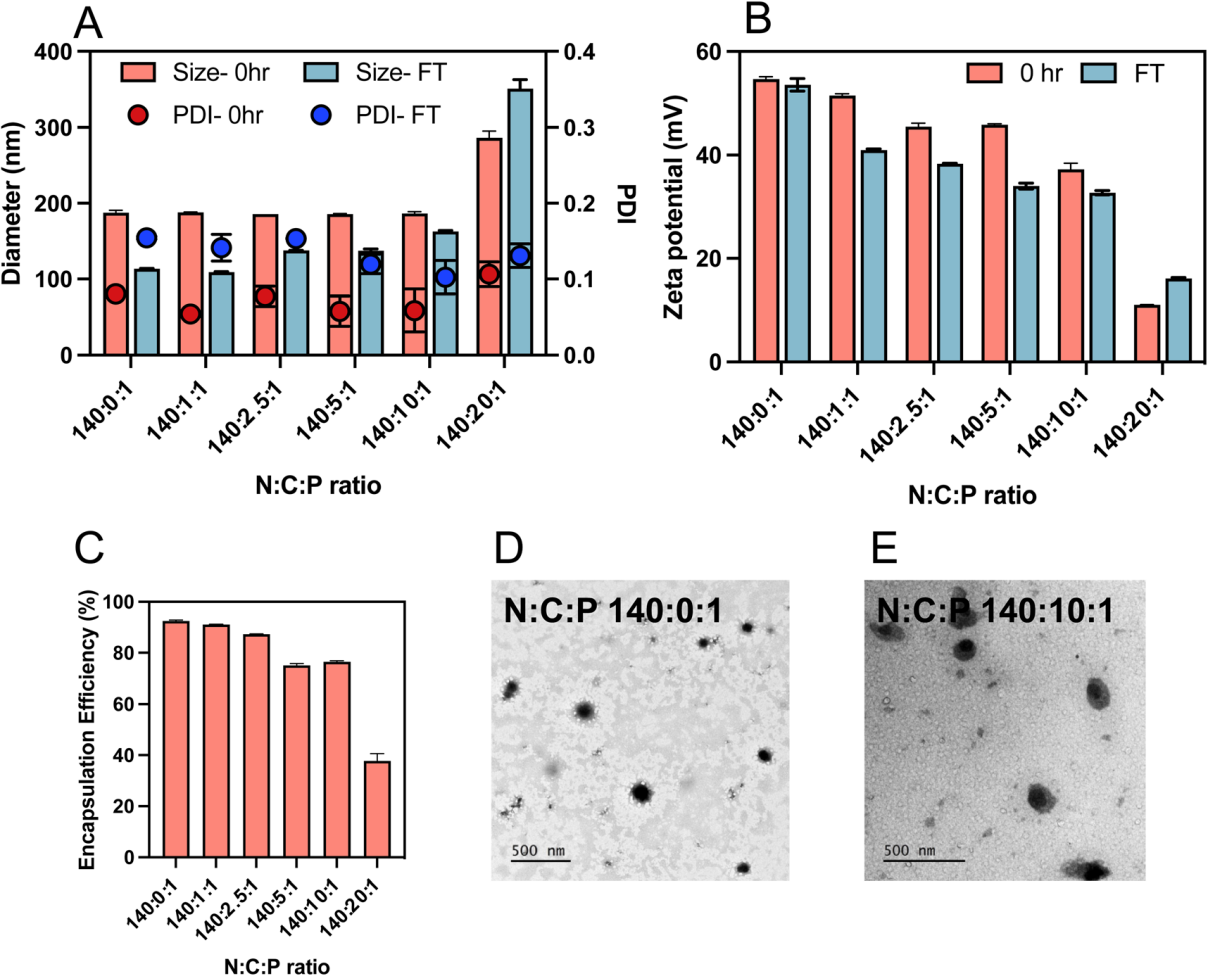
Physicochemical characterisation of ternary complexes. (A) Particle size and PDI, and (B) zeta potential of freshly prepared (0 h) and freeze-thawed (FT) ternary complexes prepared with an N : P ratio of 140 : 1 and different C ratios (0–20). (C) RNA encapsulation efficiency of ternary complexes different N : C : P ratios assessed using the RiboGreen assay. TEM of (D) polyplex (pHDDA–ABOL : saRNA = 140 : 1) and (E) ternary complex (140 : 10 : 1) which stained with 2 wt% uranyl acetate (scale bar: 200 nm).

Having identified that γ-PGA ternary complexes exhibited high transfection efficiency, we then sought to improve further this formulation by assessing the impact of PGA content on the polyplex physicochemical properties and transfection efficiency. Therefore, ternary complexes, utilising a γ-PGA content ranging from N : C : P = 140 : 0:1 to 140 : 20 : 1, were prepared using the same methodology described above. The zeta potentials of the ternary complexes decreased from 24.1 to 11 mV with increasing γ-PGA content C ratio (carboxylate ratio) from 0 to 10 (Fig. 4B). Interestingly, after adding the γ-PGA, the sizes of the ternary complexes did not change notably up to a C ratio of 10 (Fig. 4A). This indicates that incorporation of γ-PGA did not affect the interaction between the saRNA and polymer. However, there was a rapid increase in the size when the C ratio was increased from 10 to 20 (*i.e.* N : C : P = 140 : 20 : 1) which indicated the destabilisation of the ternary complexes into looser structures and aggregates.

The RiboGreen assay was used to determine the impact of γ-PGA addition on the extent of saRNA entrapment. The encapsulation of the nucleic acid decreased by adding γ-PGA (variation of N : C : P ratio from 140 : 0 : 1 to 140 : 5 : 1) and further decreased with more γ-PGA added (N : C : P ratio of 140 : 10 : 1, Fig. 4C). This entrapment reduction could be attributed to the competition between the negatively charged polymers and saRNA for electrostatic interaction with the pHDDA–ABOL polymer. Additionally, the Ribogreen dye may be able to penetrate better into the ternary complex with high amount of γ-PGA, which possesses a looser structure, resulting in lower apparent encapsulation efficacy. TEM imaging demonstrated that most of the polyplexes were approximately spherical (Fig. 4D and S2†), with some minor distortion after incorporation of γ-PGA in the ternary complexes (Fig. 4E). However, given the inherent difficulties in sample preparation for these complexes during TEM, some shape/morphology changes may be an artifact of drying. Further supplementary TEM images can be found in the ESI (Fig. S2†).

The instability of many cationic polymer/nucleic acid complexes in aqueous suspensions requires that they are prepared immediately before their administration.^33^ Therefore, the colloidal stability of the complexes after freeze-thawing was tested to elucidate the influence of γ-PGA addition. Ternary complexes with different γ-PGA amounts (C ratios from 0 to 20) were prepared and stored in the freezer for 24 h. The size, zeta potential, and PDI of the freshly prepared (0 h) and freeze-thawed (24 h) ternary complexes were measured as shown in Fig. 4A. The results indicate a decrease in size and zeta potential of all ternary complexes after freeze-thawing except for those prepared at the C ratio of 20. Notably, the data revealed a major PDI change after 24 h freeze-thawing for ternary complexes with a low amount of γ-PGA (C ratios from 0 to 5) suggesting formulation aggregation. Conversely, the PDI of ternary complexes with high γ-PGA amount (C ratios of 10 and 20) did not show significant change, indicating that the incorporation of the γ-PGA contributes to size stability and could reduce the ternary complexes aggregation.

#### *In vitro* characterisation

The effects of varying γ-PGA amount on intracellular delivery of luciferase-encoded saRNA were evaluated using four distinct cell types, *i.e.* HEK293T, moDC, NIH 3T3, and A431. *In vitro* transfection efficacy and cytotoxicity of formulations with an N : P ratio of 140 : 1 with different amounts of the γ-PGA (C ratio: 0, 1, 2.5, 5, 10, 20) containing 100 ng per well of saRNA were evaluated after 4 h incubation with the cells.

**Fig. 5:**
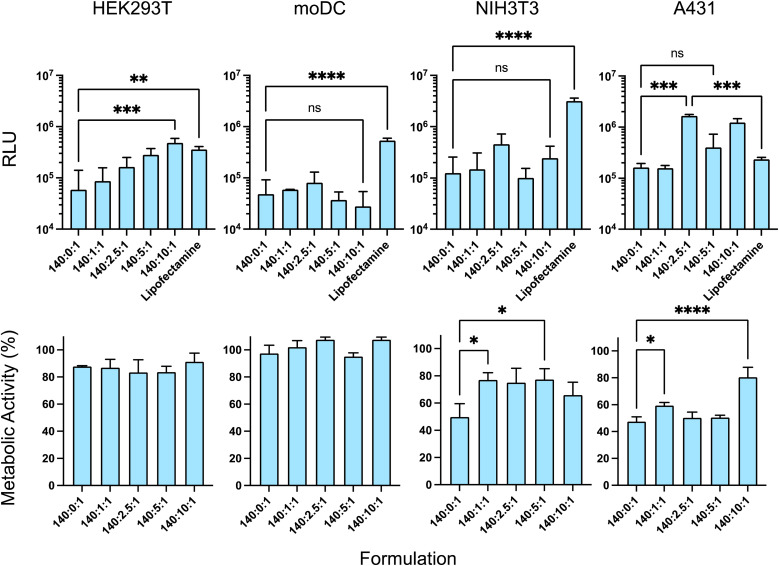
*In vitro* transfection efficacy and cytotoxicity. Luciferase expression in Relative Light Units (RLU), and cell metabolic activity was investigated in four different cell lines. HEK293T, moDC, NIH 3T3, and A431 cell lines were transfected with ternary complexes with N : P ratio 140 : 1 and different γ-PGA amounts (C ratios 0, 1, 2.5, 5, 10) for 4 h.

As HEK293T cells are known for their rapid growth rate and ease of transfection, this cell line was initially used to investigate the transfection efficacy of the formulations. In HEK293T, a significant increase in transfection occurred with an increase in the amount of γ-PGA (C ratio) from 0 to 10 and the ternary complex with the N : C : P ratio of 140 : 10 : 1 exhibited the highest transfection efficacy (Fig. 5) (note that C = 0 represents binary polyplexes, and any other C ratios represent a ternary complex).^34^

Then, different skin cell types were used relevant to future subcutaneous or intramuscular formulation administration, *i.e.* NIH 3T3 (fibroblast cell line) and moDC. Additionally, the epidermoid carcinoma cell line A431 was employed to establish the effect of the formulation effect in neoplastic skin cells. In the A431 cell line, adding the γ-PGA improved the transfection efficacy, albeit without a linear correlation to γ-PGA amount. The N : C : P ratios of 140 : 2.5 : 1 and 140 : 10 : 1 yielded the greatest transfection among the other formulations. However, in moDCs and NIH 3T3 cells, which demonstrated lower polyplex transfection efficacy compared to lipofectamine, the addition of γ-PGA did not enhance transfection, and different N : C : P ratios showed approximately similar cellular uptake (Fig. 5). Notably, the lipofectamine had relatively the same transfection efficacy in all cell lines, however, the addition of the anionic polymer had quite variable activity across all cell lines. This suggested that the transfection efficiency of the formulation depends on the target cell type, while the polymer displayed variable activity in different cell lines. These data support the concept that controlled cell and tissue targeting may require highly specific polymers to deliver saRNA effectively.

The incorporation of γ-PGA in the ternary complexes was also expected to reduce cytotoxicity as a result of the reduction in the zeta potential of the formulation. We therefore assessed the impact of γ-PGA incorporation in the formulation on cell viability. The effects of the pHDDA–ABOL:γ-PGA:saRNA ternary complexes with different γ-PGA amounts (saRNA Conc: 100 ng per well and N : C : P ratios: 140 : 0 : 1, 140 : 1 : 1, 140 : 2.5 : 1, 140 : 5 : 1, 140 : 10 : 1) on the metabolic activities of cells after 4 h incubation were studied using a PrestoBlue assay in the different cell lines, *i.e.* HEK293T, moDC, NIH 3T3, and A431. There were no significant decreases in metabolic activity toxicity when HEK293T and moDC cells were treated with the polymer–nucleic acid complexes and their metabolic activities remained around 90% (Fig. 5). However, the metabolic activities of the NIH 3T3 and A431 cells differed from those of the other cell lines after incubation with the various formulations. The polyplexes with higher surface charges reduced metabolic activities whereas incubation with the ternary complexes resulted in a significant reduction in cellular toxicity in NIH 3T3 and A431 cells. Specifically, the ternary complexes with an N : C : P ratio of 140 : 10 : 1 had the least toxicity in the NIH 3T3 cell line. Although all the formulations affected metabolic activity in NIH 3T3 and A431 cell lines, the transfection data indicated higher transgene expression per cell compared to the HEK293T and moDC cell lines. Overall, the data showed that incorporating the γ-PGA into the complexes either improved the transfection efficacy or metabolic activity, depending on the cell types.

**Fig. 6:**
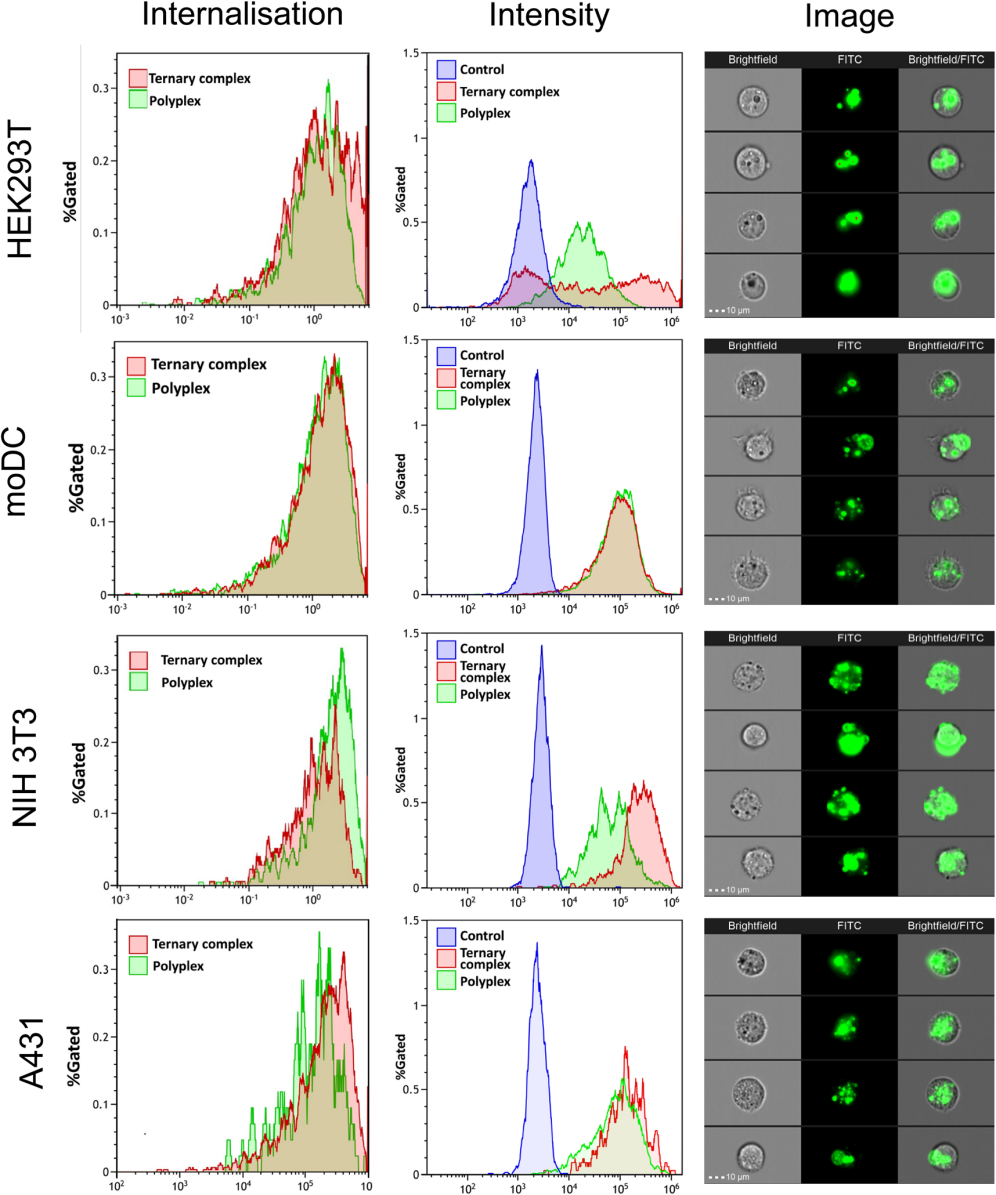
Flow cytometry analysis internalization percentage and fluorescence intensity of FITC-labelled pHDDA–ABOL:PGA:saRNA complexes with N : C : P ratios of 10 : 0 : 1 and 140 : 10 : 1 were measured by flow cytometry after 4 h incubation in different cell lines (HEK293T, moDC, NIH3T3, and A413). Imaging flow cytometry images showing the presence of the ternary complexes in the cells (overlap of the brightfield and FITC channels).

The internalisation of the complexes into cells was assessed using flow cytometry, while confocal microscopy was used to visualize the intracellular delivery of saRNA using FITC-labelled formulations. As shown in Fig. 6, the FITC-labelled formulations entered all the cell lines within 4 h (internalisation >60%). For HEK293T and NIH3T3 cell lines, the ternary complexes demonstrated higher FITC fluorescence intensity compared with the polyplexes, whereas there were no significant differences for the moDC and A431 cells. These data indicated that internalisation was rapid for all the complexes into all the cells, but the extent of nanoparticle entry varied across the cell lines, in line with the observation that transfection efficacy was also cell-line dependent.

## Discussion

In this study we aimed to evaluate the effects of added polyanions to polycation/saRNA complexes, as prior studies had shown that mixed polycation/polyanion systems could transfect certain cancer cells *in vitro* and *in vivo* with greater efficacy for pDNA and with siRNA than the binary polycation nucleic acid complexes alone.^35^ For assays with saRNA we made the specific choices of poly(β-amino-ester)s (PBAEs) as the polycations as these have been shown to be effective delivery agents for saRNA in pre-clinical studies,^36^ and poly(glutamic acid)s (PGAs) owing to their known safety profile and ready availability.^37–39^ The versatility of the routes to PBAEs and the ease of end-group modification *via* the final capping stage has been exploited by many groups, with a range of chemistries adopted and with or without cell-targeting agents.^40–42^ In addition, Green *et al.* have shown that the end-groups of PBAEs can modulate transfection activity of delivered nucleic acids in a range of cell lines,^43^ while substitution of more hydrophobic regions in the PBAE backbone can alter biodistribution and efficacy *in vivo*.^44^ For these studies, we decided to end-cap with 4-aminobutanol, in order to retain the amine-containing functional unit from the backbone into the polymer termini, as the central experimental variable for this study was the effect of the added polyanion (PGA) rather than the PBAE end-groups or any targeting agents. The PBAEs synthesised were of relatively low molar mass (∼8400 g mol^−1^) and thus very much smaller than the saRNA (∼9000 nt, >3 000 000 g mol^−1^), hence high polycation : RNA ratios were needed to condense the long polyanions sufficiently to form nanoparticles of sizes likely to enter target cells. At N : P ratios of 70 : 1 and 140 : 1, the binary complexes were of ∼200 nm diameter, with zeta potentials of ∼+20 mV and greater than 90% incorporation of saRNA. There was a notable increase in particle zeta potentials for binary and formulations produced in principle with the same N : C : P 140 : 0 1 ratio composition but using a 1 : 1 (+20 mV, Fig. 2C) volume ratio or a 2 : 1 volume ratio (+60 mV, Fig. 5B), suggesting that the arrangement of the polycation may be dependent on formulation process.

Addition of the PGAs after complexation, in effect ‘coating’ the preformed PBAE/saRNA complexes, resulted in formulations which were mostly larger and more polydisperse than complexes prepared by mixing PBAE, PGA and RNA to form ternary complexes. Initial transfection assays in HEK293T cells indicated higher transgene expression with one of the ternary complexes compared to its ‘coated’ analogue, and no significant differences with two other formulations, but all were more effective in this cell line than the positive control lipofectamine. However, the increases in polydispersity for the coated complexes implied a reduction in colloidal stability, and thus we took forward the ternary complexes for further experiments to test the effects of freeze–thaw cycles, which are critical attributes of commercial formulations. All the ternary complexes were stable to freeze–thaw conditions, as determined by particle size and polydispersity measurements, with either a slight reduction or no marked change in either parameter except for the formulation with the highest PGA content. A second key criterion for a formulation is that it is non-toxic, and as shown in Fig. 5, while all the complexes were well tolerated in HEK293T and moDC cells, for NIH3T3 and A431 cells, the PBAE/saRNA complexes were toxic but the ternary systems were not, with significant differences in observed metabolic activities. Fig. 5 also shows that for HEK293T and A431 cells, some of the ternary complexes were more effective than the binary polycation/saRNA or lipofectamine saRNA in luciferase transfection. Flow cytometry analysis exhibited a significant increase in median fluorescence intensity upon incubation of HEK293T cells with the ternary complexes compared to the binary polyplexes. In terms of transfection, the effect of added γ-PGA was most significant in the A431 cells, in which the ternary complexes outperformed both PBAE/saRNA and lipofectamine/saRNA.

These data suggested that the transfection efficacy of the polyplexes was enhanced in some cells by the presence of the specific negatively charged polymer, *i.e.* γ-PGA. This might be attributed to the distinctive mechanisms by which γ-PGA enters certain cells.^45^ Previous studies have reported that γ-PGA enhances the release of RNA trapped within ternary complexes, but also may facilitate cellular uptake through the γ-glutamyl transpeptidase membrane protein (GGT).^34,46,47^ This enzyme also expressed by certain epithelial neoplasms.^48,49^ Therefore, we believe that the improved transfection observed in A431 cells, *i.e.* epidermoid carcinoma cells, is related to the expression of the GGT enzyme by neoplastic epithelial cells. One other factor increasing transfection for the ternary systems might be enhanced endosomal escape, as shown for the mixed systems of Hsu *et al.* previously. The γ-PGA component in the ternary complexes might be expected to undergo some protonation at the lower pH conditions of late endosome/early lysosomes, which in turn could lead to membrane disruption *via* insertion of uncharged polymer chains. Attempts to evaluate this *via* confocal microscopy were not conclusive, as while images (Fig. S1†) confirmed that the polymer–RNA complexes were rapidly internalised, full quantification of Lysotracker dye relative to the FITC-label on the PBAE components was not possible due to the pH-sensitivity of the FITC-dye and the variations in pH as complexes pass through endolysosomal regions. Nevertheless, the combination of the PBAE backbone and the γ-PGA was clearly potent in respect to saRNA transfection in the A431 cell line in particular, and the fact that these complexes were stable to freeze-drying suggests some promise prior to *in vivo* studies.

## Conclusions

In this paper we have described the development of self-assembled polycation/RNA/poly(glutamic acid) ternary complexes, in which the role of the PGA is to reduce the potential toxicity of the cationic components and enhance the transfection efficacy for saRNA delivery. The formation of the ternary complexes was experimentally facile and was accompanied by a decrease in the zeta potentials of the resultant particles compared to their polycation/RNA analogies, while having no significant impact on the size and RNA entrapment efficacy. The findings further demonstrated that the *in vitro* RNA transgene expression from the ternary complexes depended on the formulation parameters, including the N/P ratio, type, and molar mass of the PGA, as well as the specific cell lines used. The highest levels of transfection were found in HEK293T and moDC cells, and indication of cytotoxicity were reduced in NIH 3T3 and A431 cell lines by the introduction of the PGA into the polycation/RNA complexes. Furthermore, the high molar mass γ-PGA ternary complexes outperformed the low molar mass α-PGA complexes in the induction of luciferase expression. Based on the improved transfection efficacy and reduced toxicity observed with this formulation, we anticipate that pHDDA–ABOL:saRNA:γ-PGA could be an efficient saRNA delivery system. Moreover, given the known immunostimulatory properties of γ-PGA as a vaccine adjuvant,^50–52^ these formulations may be promising for future applications in vaccination. Furthermore, the elevated GGT expression observed in tumour cells, which contributes to drug resistance, might serve as a crucial factor for enhancing cellular transfection efficacy in PGA-containing formulations. Exploiting this unique characteristic, our ternary complex holds potential as a novel approach for tumour treatment and effectively overcoming drug resistance mechanisms.^53^ However, future studies are warranted to evaluate the effectiveness of these formulations *in vivo*.

## Data availability

All relevant data can be obtained upon request from the authors at cameron.alexander@nottingham.ac.uk and p.gurnani@ucl.ac.uk.

## Conflicts of interest

RJS is a founder of VaxEquity Ltd and PFM is a co-founder and employee of VaxEquity Ltd. VaxEquity Ltd was not involved in these studies.

## Supplementary Material

NA-006-D3NA00794D-s001
