# Supplementary material for: Charge neutralized poly(β-amino ester) polyplex nanoparticles for delivery of self-amplifying RNA
Source: Nanoscale Adv. 2024 Jan 24;6(5):1409–22. doi: 10.1039/d3na00794d (PMC10898429; doi:10.1039/d3na00794d)
Supplement: NA-006-D3NA00794D-s001 [file NA-006-D3NA00794D-s001.pdf]

Electronic Supplementary Material (ESI) for Nanoscale Advances.
This journal is © The Royal Society of Chemistry 2024

Charge neutralized poly(β-amino ester) polyplex nanoparticles for delivery of self-amplifying RNA

Nazgol Karimi Dastgerdi,[ab] Nurcan Gumus,[a] Hulya Bayraktutan,[a] Darryl Jackson,[c] Krunal Polra,[d] Paul F. McKay,[d] Fatemeh Atyabi,[b] Rassoul Dinarvand,[be*] Robin J. Shattock,[d] Luisa Martinez-Pomares,[c] Pratik Gurnani,[f*] Cameron Alexander[a*]

Supporting Information

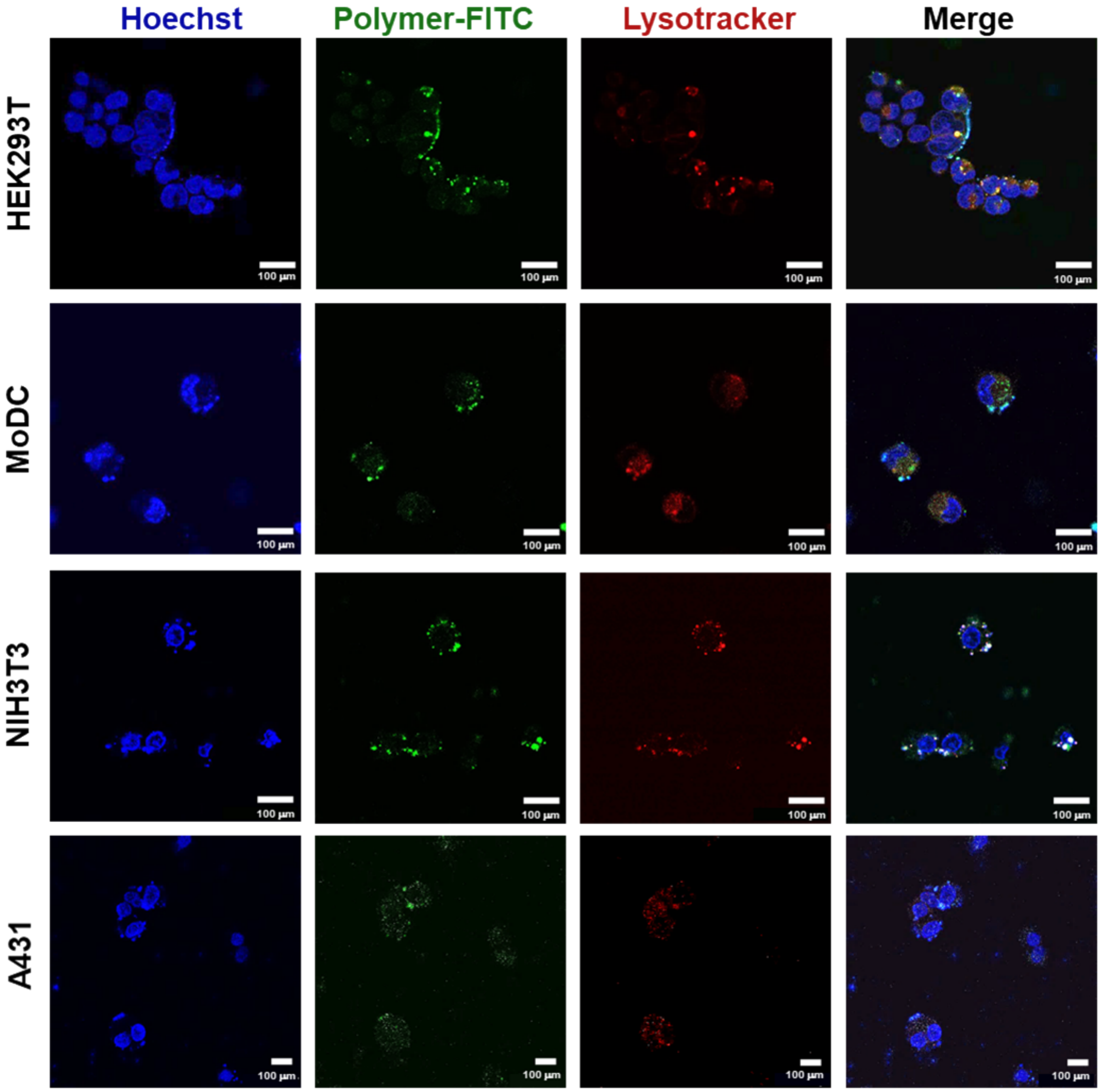

Figure S1. A) Confocal laser scanning microscopy images of different cell lines (HEK293T, moDC, NIH3T3, and A431) after 4 hr incubation with FITC labelled ternary complexes with N:C:P ratio140:10:1. The nuclei were stained with Hoechst 33342 (blue) and lysosomes were stained with Lysotracker Red. Scale bar =100 µm. Images have enhanced contrast for greater clarity.

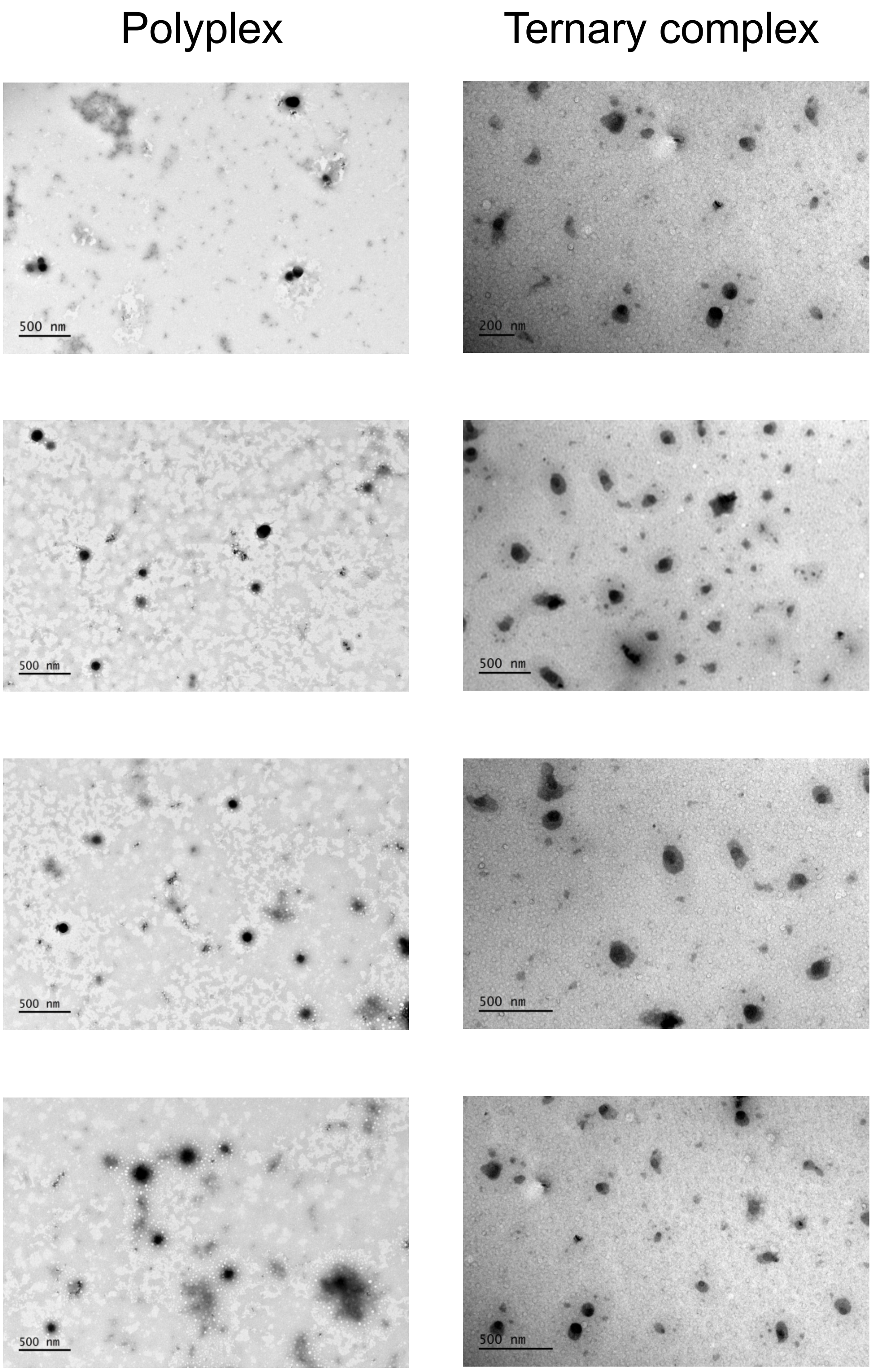

Figure S2. Transmission electron micrographs of PBAE-RNA polyplexes and PBAE-γ-PGA-RNA ternary complexes.
